# Use of Saliva Analytes as a Predictive Model to Detect Diseases in the Pig: A Pilot Study

**DOI:** 10.3390/metabo15020130

**Published:** 2025-02-13

**Authors:** Eva Llamas-Amor, Alba Ortín-Bustillo, María José López-Martínez, Alberto Muñoz-Prieto, Edgar García Manzanilla, Julián Arense, Aida Miralles-Chorro, Pablo Fuentes, Silvia Martínez-Subiela, Antonio González-Bulnes, Elena Goyena, Andrea Martínez-Martínez, José Joaquín Cerón, Fernando Tecles

**Affiliations:** 1 Interdisciplinary Laboratory of Clinical Analysis (Interlab-UMU), Veterinary School, Regional Campus of International Excellence ‘Campus Mare Nostrum’, University of Murcia, Campus de Espinardo, 30100 Murcia, Spain; eva.llamasa@um.es (E.L.-A.); alba.ortinb@um.es (A.O.-B.); mariajose.lopez28@um.es (M.J.L.-M.); alberto.munoz@um.es (A.M.-P.); silviams@um.es (S.M.-S.); jjceron@um.es (J.J.C.); 2 Pig Development Department, Moorepark Animal and Grassland Research Centre, Teagasc, Irish Agriculture and Food Development Authority, P61 C996 Cork, Ireland; egmanzanilla@gmail.com; 3 School of Veterinary Medicine, University College Dublin, D04 W6F6 Dublin, Ireland; 4 Institute for Biomedical Research of Murcia, IMIB-Arrixaca, 30120 Murcia, Spain; julian-jesus.arense@um.es; 5 Anatomy and Compared Pathology Anatomy Department, Veterinary School, Regional Campus of International Excellence ‘Campus Mare Nostrum’, University of Murcia, Campus de Espinardo, 30100 Murcia, Spain; aida.miralles@um.es; 6 Cátedra Universitaria Grupo Fuertes, 30100 Murcia, Spain; pablo.fuentes@cefusa.com; 7 Departamento de Producción y Sanidad Animal, Facultad de Veterinaria, Universidad Cardenal Herrera-CEU, CEU Universities, C/Tirant lo Blanc, 7, 46115 Valencia, Spain; antoniogbulnes@cuartesa.com; 8 Cuarte S.L. Grupo Jorge, Ctra. De Logroño, Km 9.2, 50120 Zaragoza, Spain; 9 Animal Health Department, Veterinary School, Regional Campus of International Excellence ‘Campus Mare Nostrum’, University of Murcia, Campus de Espinardo, 30100 Murcia, Spain; goyena@um.es; 10 Agropecuaria Casas Nuevas S.A., Ctra. de las Palas, Fuente Álamo, 30320 Murcia, Spain; amartinez@grupo-frances.es

**Keywords:** biomarkers, infectious disease, swine, oral fluid

## Abstract

Background/Objectives: Saliva is gaining importance as a diagnostic sample in pigs. The aim of this research was to evaluate a panel of salivary analytes in three porcine diseases and establish predictive models to detect them. Methods: Saliva samples were obtained from healthy pigs (*n* = 97) and pigs affected by meningitis due to *Streptococcus suis* (*n* = 118), diarrhea due to enterotoxigenic *Escherichia coli* (ETEC, *n* = 77), and porcine reproductive and respiratory syndrome (PRRS, *n* = 52). The following biomarkers were analyzed: adenosine deaminase (ADA), haptoglobin (Hp), calprotectin (Calp), aldolase, alpha-amylase (sAA), lactate dehydrogenase (LDH), total protein (TP), and advanced oxidation protein products (AOPPs). Predictive models based on binary logistic regression and decision trees combining those analytes for detecting specific diseases were constructed. Results: The results showed a different biomarker profile between the groups. *S. suis* and ETEC pigs showed higher values of ADA, Hp, Calp, aldolase, sAA, LDH, and TP than healthy pigs. Pigs with PRRS showed higher values of Hp, Calp, sAA, and LDH than healthy animals. The constructed predictive models showed overall accuracies of over 78% and 87% for differentiating ETEC and PRRS, respectively, whereas the models did not accurately predict *S. suis* infection. Conclusions: Salivary analytes show different changes in pigs depending on the disease, and the combination of these analytes can contribute to the prediction of different diseases. Further studies should be conducted in larger populations to confirm these findings and evaluate their possible practical applications.

## 1. Introduction

Saliva has gained great importance in recent years as a biological sample for detecting infectious diseases in pigs. For instance, the enzyme-linked immunosorbent assay (ELISA) and polymerase chain reaction (PCR) have been used in saliva for the detection of porcine epidemic diarrhea virus (PEDV) [1] and porcine reproductive and respiratory syndrome virus (PRRSV) [2,3] infections. In addition to specific tests, salivary biomarkers can be used as non-specific indicators of health status, and some of them can be used for detecting inflammation or sepsis. For example, acute-phase proteins can be measured in saliva, and increases are detected in inflammation. In addition, biomarkers of sepsis could be measured in saliva, helping to detect sepsis and contributing to a more appropriate use of antibiotics [4]. There are still no standardized protocols for saliva collection in pigs. Since the way in which the samples are obtained could have relevance in the results obtained, studies are currently being carried out in order to optimize the method of obtaining saliva samples in this species [5].

Three infectious diseases that are of high importance in pigs are meningitis due to *Streptococcus suis*, diarrhea due to *Escherichia coli*, and pneumonia or abortions due to PRRSV. *S. suis* is responsible for important economic losses [6,7]. Meningitis is the main clinical manifestation associated with *S. suis*, but it can produce arthritis, pneumonia, or endocarditis [8]. *S. suis* is also a zoonotic pathogen [9], which makes early identification of the disease a key feature. Enterotoxigenic *E. coli* (ETEC) is another common infectious pathogen affecting piglets. Diarrhea is its main clinical manifestation [10], but it can progress to a septic condition [11]. PRRS is a viral infection that causes reproductive failure in pregnant sows and acute respiratory illness in growing pigs [12,13] and also produces immune suppression [14], allowing other pathogens to cause secondary infections as part of the Porcine Respiratory Complex.

Previous studies have shown that salivary biomarkers are affected in pigs with *S. suis* and *E. coli* infections. Namely, biomarkers of immunity, such as adenosine deaminase (ADA), biomarkers of inflammation such as haptoglobin (Hp) and total protein (TP), biomarkers of sepsis such aldolase [15], biomarkers of stress such as salivary alpha-amylase (sAA), and biomarkers of redox status such as advanced oxidation protein products (AOPPs) increase in these diseases [16].

Machine learning (ML) is a branch of artificial intelligence that builds algorithms to find and predict relationships between different variables [17,18]. This computational approach has demonstrated potential use in farm animal production for several purposes, constructing algorithms capable of identifying predisposing factors for infectious diseases as well as their prevention. For example, predisposing factors have been identified for lameness [19], pneumonia [20], or tail biting [21] in growing pigs. Also, variables useful for the prevention of PRRS have been identified [22], contributing to better control of disease transmission [23]. In the diagnosis of diseases, an ML approach using a sound recording system was developed for the detection of respiratory diseases [24]. The diagnosis of specific diseases by ML has been attempted in companion animals such as dogs, where algorithms using clinical and laboratory data have been useful for the development of a predictive model for the diagnosis of hypoadrenocorticism [25] or Cushing’s disease [26]. To the authors’ knowledge, no methods based on ML have been found for the development of predictive models for detecting porcine diseases.

The aim of this report was to evaluate the possible differences observed in a panel of salivary analytes in three different porcine diseases (meningitis due to *S. suis*, diarrhea due to ETEC, and respiratory or reproductive issues due to PRRSV infection) and establish predictive models to differentiate the diseases. This allows for gaining knowledge about the dynamics of analytes in saliva in infectious diseases of pigs and also for exploring if some of the analytes, as well as their analyses through ML techniques, could be helpful in developing predictive models as complementary tools for the detection of diseases.

## 2. Materials and Methods

### 2.1. Animals

A total of 344 pigs [(*Sus scrofa domesticus*) (Landrace × Large White)] in the post-weaning period from different farms located in southern Spain were included in this study. According to European legislation (Council Directive 2001/88/CE of 23 October 2001 amending Directive 91/630/CEE concerning minimum standards for the protection of pigs), all animals were housed in pens containing standard dry feeders and nipple drinkers to provide ad libitum access to feed and water, with a minimum space of 0.24 m^2^ per animal. Pigs in the nursery phase received a starter diet containing 2.52/kg Mcal/Kg net energy (NE) and 172.2 g/kg crude protein (CP). Pigs in the growing phase received a diet containing 2.45 Mcal/Kg NE and 164 g/kg CP. All animals were vaccinated against *Mycoplasma hyopneumoniae* (Stellamune Mycoplasma, inactivated *Mycoplasma hyopneumoniae* NL 1042, Pfizer Animal Health, Madrid, Spain) and porcine circovirus type 2 (Porcilis^®^ PCV, MSD Animal Health, Boxmeer, The Netherlands) at weaning. Diseased animals were sampled before any treatment was administered in order to avoid interferences in laboratory measurements.

The animals were divided into the following groups:

(a) Pigs with meningitis due to *S. suis* infection (*n* = 118): Animals included in this group were between 6 and 11 weeks old (mean 7.78, SD 2.40). The pigs had clinical signs compatible with *S. suis* infection, such as ataxia, anorexia, lateral recumbency, and padding [27]. Infection was confirmed by the bacterial isolation and characterization [28] of *S. suis* serotype 9.
(b) Pigs with diarrhea due to ETEC (*n* = 77): All animals were 6 weeks old. The animals included in this group showed moderate to severe clinical signs including diarrhea, lethargy, growth retardation, and dehydration. The diagnosis was confirmed by the detection of *E. coli* F4 and heat-labile toxin in fecal samples obtained with rectal swabs, as previously described [29].
(c) Pigs with PRRS (*n* = 52): The pigs were between 8 and 9 weeks old (mean 8.40 and SD 0.50). These animals were from farms with a history of PRRSV infection, and all of them showed diverse clinical symptoms including loss of body condition; sunken flanks; a rough, hirsute coat; lethargy; and mild dyspnea. In this group, the rectal temperature was evaluated, and none of the pigs showed hyperthermia, which would be compatible with the absence of concomitant infections. The final diagnosis was confirmed by the detection of the European strain of PRRSV in the blood using a PCR kit (Roche, Mannheim, Germany) or specific antibodies with an ELISA kit (IDEXX Laboratories, Westbrook, ME, USA).
(d) Healthy animals (*n* = 97): The pigs in this group were between 6 and 13 weeks old (mean 9.98 and– SD 2.54). This group included clinically healthy pigs with no symptoms of disease obtained from the Veterinary Teaching Farm of the University of Murcia, which is a PRRS-free farm.

The animal study protocol was approved by the Institutional Review Board (or Ethics Committee) of the University of Murcia (protocol code A13220196; date of approval 4 March 2021).

### 2.2. Saliva Sampling

Saliva samples were collected by allowing the pigs to chew a 5 × 2 × 2 cm piece of a polypropylene sponge (Esponja Marina, La Griega E. Koronis, Madrid, Spain) clipped to a metal rod. Only one animal was allowed to chew each piece of sponge in order to obtain individual samples. When each piece of sponge was thoroughly moistened, it was placed into a Salivette tube (Sarstedt, Aktiengesellschaft & Co., Nümbrecht, Germany) and maintained refrigerated in a box with ice until arrival at the laboratory, which took place in two hours at the most. Thus, one sponge was used for only one animal. Once at the laboratory, the Salivette tubes containing the sponges with saliva samples were centrifuged at 3000× *g* and 4 °C for 10 min. Saliva samples contaminated by debris or feed particles or those with a brownish or reddish color were discarded. Saliva was then collected and stored at −80 °C in Eppendorf tubes until analysis. All samples were obtained and stored within a period of two months and then analyzed in a single batch in order to reduce variability.

### 2.3. Laboratory Analyses

The biomarkers detailed below were analyzed in the saliva.

#### 2.3.1. Inflammation and Immunity Biomarkers

The enzymatic activity of ADA was estimated by a commercially available spectrophotometric assay (Adenosine Deaminase assay kit, Diazyme Laboratories, Poway, CA, USA). Hp was measured by an in-house assay based on the AlphaLISA method. Calprotectin (Calp) was determined by the BÜHLMANN fCal Turbo^®^ assay (BÜHLMANN, Laboratories AG, Schönenbuch, Switzerland). Aldolase activity was determined through a commercially available reagent kit (Aldolase, Randox Laboratories Ltd., Crumlin, UK).

#### 2.3.2. Stress Biomarkers

Alpha-amylase (sAA) activity was evaluated using a commercial colorimetric test (Alpha-Amylase, Beckman Coulter Inc., Fullerton, CA, USA).

#### 2.3.3. Tissue Damage Biomarkers

The enzymatic activity of lactate dehydrogenase (LDH) was measured with commercial tests from Beckman (Beckman Coulter Inc., Fullerton, CA, USA). TP was analyzed using a commercial colorimetric kit for urinary and cerebrospinal fluid protein measurement (Protein in Urine and CSF, Spinreact, Barcelona, Spain).

#### 2.3.4. Biomarkers of Redox Status

The concentration of AOPPs in the saliva was determined using a previously described method, in which Chloramine-T was used as a calibrator and the change in absorbance was recorded at 340 nm in the presence of potassium iodide in acidic conditions [30].

ADA, sAA, calprotectin, LDH, TP, aldolase, and AOPPs were analyzed in the Olympus AU600 biochemistry autoanalyzer (Olympus AU600, Olympus Diagnostica GmbH, Hamburg, Germany). Hp was determined in a 96-well plate reader (PerkinElmer, Inc., Waltham, MA, USA). All assays have been previously validated and used in porcine saliva samples [16].

### 2.4. Statistical Analysis

Graphical methods and the Shapiro–Wilk test were used to assess the distribution of the data, giving a non-parametric distribution. After that, differences between groups were assessed by a Kruskal–Wallis test followed by Bonferroni pairwise comparison. The size effect (*r*) was calculated for each pairwise comparison.

To differentiate each pathological condition (*S. suis* infection, ETEC, or PRRS) from the others and the healthy pigs, receiver operating characteristic (ROC) analysis and decision trees (DTs) were used. For this purpose, animals with each specific disease were compared to the rest of the animals included in the study. Firstly, ROC curves were generated individually for the different analytes in order to know whether they had any value in discriminating one diseased group from the rest of the pigs. Those analytes showing a significant area under the curve (AUC) were selected for calculating cut-off values according to previously described methods [31]. The sensitivity, specificity, and overall accuracy were calculated from the ROC analyses. Then, stepwise binary logistic regression (BLR) was performed on those analytes showing a significant AUC. Those analytes included in the equation were combined for an ROC analysis in order to construct a predictive model using the cut-off values previously stated. The models were constructed with 70% of the data (training set). Then, the selected cut-off values were validated with the remaining 30% of the data (validation set). To avoid a confounding effect due to the different ages of the pigs, the age of every individual (in weeks) was considered as a covariate in the analyses.

Three decision tree (DT) models were developed using the Gini index as the splitting criterion and were pruned based on the complexity parameter through 10-fold cross-validation. Surrogate splits were implemented to manage missing data. No additional adjustments were made, except for setting the maximum depth of the final tree in the PRRS tree model. The final models were selected based on parsimonious criteria and evaluated using a test set.

All statistical analyses were performed by using a spreadsheet (Excel 2000, Microsoft Corporation, Redmond, WA, USA) and the commercial statistics package SPSS (IBM SPSS Statistics for Windows, Version 28.0. IBM Corp., Armonk, NY, USA). The decision tree analysis was conducted using the rpart (v.4.1.23) [32] and rpart.plot (v.3.1.2) [33] packages in R (v.4.4.2). The alpha level for significance was set at 0.05.

## 3. Results

**Figure 1:**
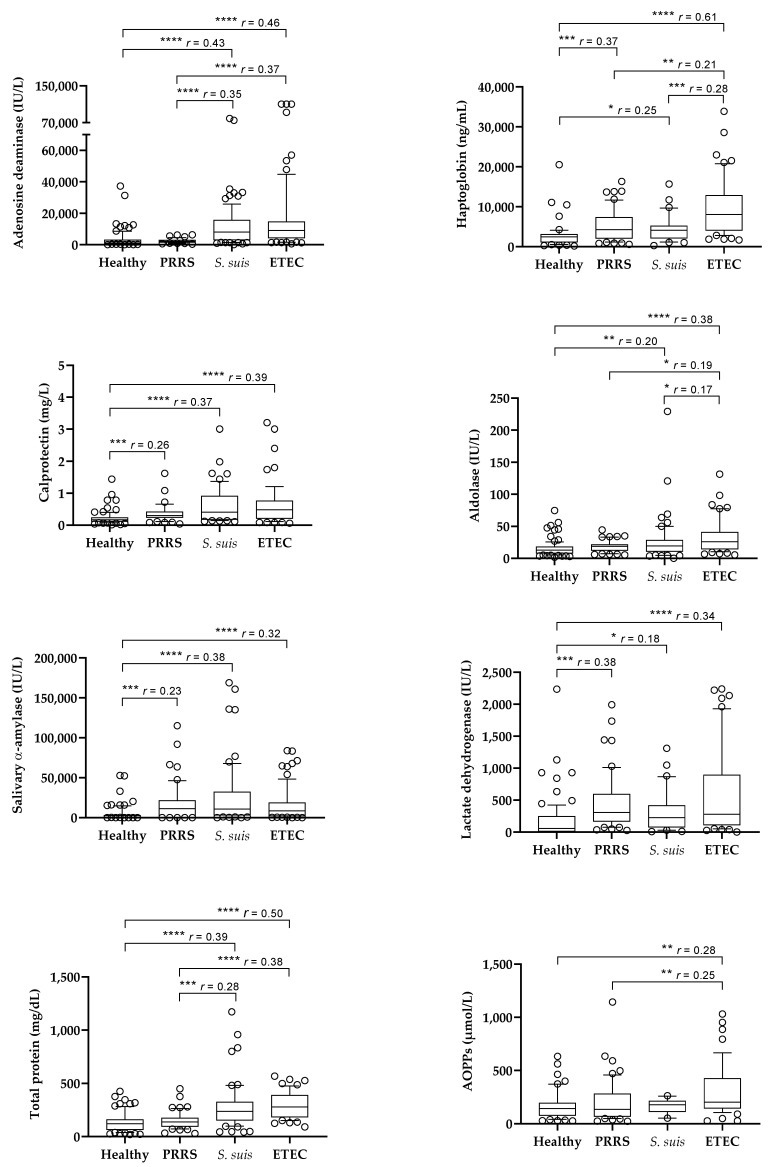
Box plots of the salivary levels of salivary biomarkers in healthy pigs (*n* = 97) and in pigs with porcine reproductive and respiratory syndrome (PRRS, *n* = 52), *Streptococcus suis* infection (*n* = 118), and enterotoxigenic *Escherichia coli* infection (ETEC, *n* = 77). Statistical analysis: asterisks indicate significant differences (* *p* < 0.05; ** *p* < 0.01; *** *p* < 0.001; **** *p* < 0.0001); *r*: size effect. AOPPs: advanced oxidation protein products.

The results of the salivary analytes obtained in the different groups of animals are shown in Figure 1. Pigs affected by *S. suis* and ETEC infections showed higher values of ADA, Hp, Calp, aldolase, sAA, LDH, and TP than healthy pigs. Pigs with PRRS showed higher values of Hp, Calp, sAA, and LDH than healthy pigs, whereas ADA, aldolase and TP were not increased.

When pigs with different diseases were compared, those with ETEC showed higher values of Hp and aldolase than pigs with *S. suis* infection and higher values of ADA, Hp, aldolase, TP, and AOPPs than the pigs affected by PRRS. Pigs with *S. suis* infection showed higher values of ADA and TP than the animals with PRRS, with no differences in other analytes between both groups. Calp, sAA, and LDH did not show differences between the groups of diseased animals.

**Figure 2:**
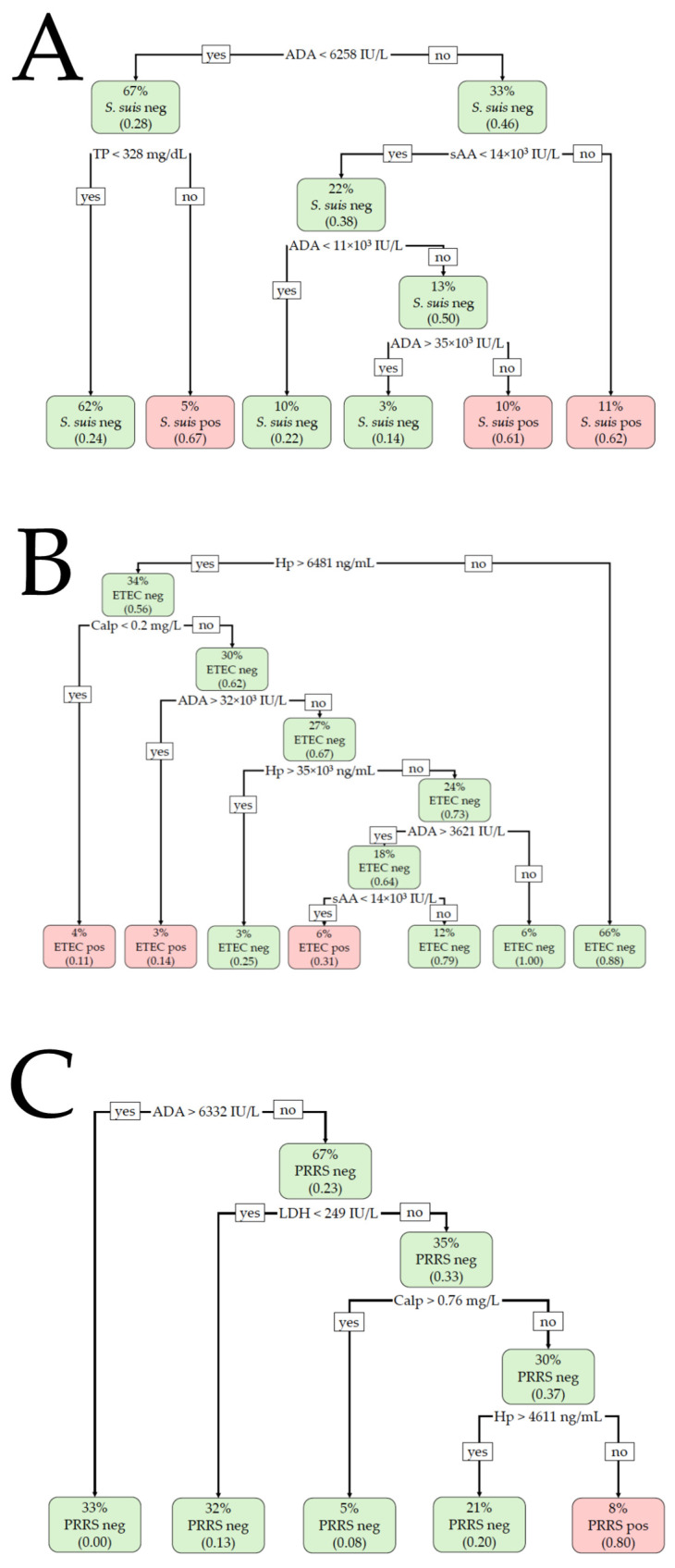
Decision tree (DT)-based classification for (**A**) *Streptococcus suis* (*S. suis*), (**B**) enterotoxigenic *Escherichia coli* (ETEC), and (**C**) porcine reproductive and respiratory syndrome (PRRS) infections. The percentage value indicates what percentage of the total animals are included within each node. The number in parentheses indicates the proportion of the animals included in each node that is positive or negative, with 0.00 being equal to 0% and 1.00 being equal to 100%. Neg: negative for the disease; Pos: positive for the disease.

The results of the BLR and DT analyses are shown in Table 1 and Figure 2. For the detection of *S. suis* infection, the BLR model included Calp and TP, providing a Nagelkerke R^2^ of 0.27 (χ^2^ = 27.0, *p* < 0.001) for the training set and an R^2^ of 0.09 (χ^2^ = 2.7, *p* = 0.261) for the validation set. Calp > 0.4 mg/L and TP > 180.83 mg/dL were suggestive of *S. suis* infection. The model did not correctly classify the animals with or without infection in the validation set, providing a non-significant AUC of 0.676 (*p* = 0.135) and an overall accuracy of 64.7%. The DT model included ADA, sAA, and TP in the training set, with ADA being the most important variable. ADA activity >6258 IU/L, together with sAA activity >14,000 IU/L, was compatible with *S. suis* infection. With an ADA value below the formerly indicated cut-off, TP > 328 mg/dL was also suggestive of a positive *S. suis* infection. The overall accuracy was 71.9% (AUC 0.668). When the model was applied to the validation set, a non-significant AUC (0.603, *p* = 0.068) was obtained and the overall accuracy was 68.6%.

For ETEC detection, the BLR model included the analytes ADA and Hp in the training set. The cut-off values were >4203.2 IU/L and >3426.69 ng/mL, respectively, for ADA and Hp. The R^2^ was 0.46 (χ^2^ = 54.9, *p* < 0.001), with an AUC of 0.852 and an overall accuracy of 80.4%. The BLR model provided an R^2^ of 0.53 (χ^2^ = 27.3, *p* < 0.001), a significant AUC of 0.884, and an overall accuracy of 81.8% when applied to the validation set. The DT model included Hp, Calp, ADA, and sAA in the training set, with Hp being the most important variable. A salivary Hp concentration >6481 ng/mL, together with Calp < 0.2 mg/L or ADA > 32,000 IU/L, was indicative of ETEC with an overall accuracy of 86.3% and an AUC of 0.788. The DT model showed an accuracy of 78.6% and an AUC of 0.682 in the validation set.

For PRRS detection, the BLR model was constructed with the analytes ADA and LDH in the training set, with cut-offs of <6332.13 IU/L and >273.6 IU/L, respectively. The R^2^ was 0.44 (χ^2^ = 49.9, *p* < 0.001), with a significant AUC of 0.838 and 75.5% accuracy. When applied to the validation set, the Nagelkerke R^2^ was 0.56 (χ^2^ = 30.7, *p* < 0.001), with a significant AUC of 0.910 and an overall accuracy of 89.6%. The DT model included the variables ADA, LDH, Calp, and Hp when applied to the training set. The most important variable was LDH, followed by Calp and ADA. Lower values of ADA and Calp (<6332.0 IU/L and <0.76 mg/L, respectively) with increased LDH and Hp (>249.0 IU/L and >4611.0 ng/mL, respectively) were compatible with PRRSV infection. The overall accuracy of the model was 89.7% (AUC 0.838). When the model was applied to the validation set, the overall accuracy was 87.3% (AUC 0.803).

## 4. Discussion

In this research, the changes in salivary biomarkers in different pathologic conditions in swine were studied. Also, the potential of those biomarkers to be used in predictive models to detect and differentiate diseases was evaluated by BLR and DT analyses. We considered two bacterial diseases, *S. suis* and *E. coli* infections, for two main reasons. Firstly, they hold clinical and economic importance. *S. suis* is one of the most important swine pathogens, producing high mortality and morbidity on pig farms [34], as well as being considered a zoonosis [35]. Also, ETEC is another common pathogen affecting piglets that is associated with a high rate of mortality [10,11]. Both diseases exert a severe economic impact on the swine industry [7]. Secondly, antibiotics are used for treating both diseases [36]. Knowing when antibiotics are necessary is one of the most challenging aspects in avoiding their unnecessary use and the subsequent development of multidrug-resistant bacteria [37,38]. In addition, a viral disease, PRRS, was also included in this research. PRRS is a major disease that induces respiratory symptoms in pigs, along with reproductive issues in sows, and causes severe economic losses worldwide [39].

Analytes related to inflammation and immunity (ADA, Hp, and Calp), sepsis (aldolase), stress (sAA), tissue damage (LDH and TP), and redox status (AOPP) were measured to obtain a wide panel of biomarkers. With the exception of Hp, the rest of the analytes are easily automatable for a fast and accurate measurement. In the bacterial diseases (*S. suis* and ETEC), pigs showed higher levels of ADA, Hp, Calp, aldolase, sAA, LDH, and TP than healthy animals. Increases in salivary inflammatory markers have been previously described in these diseases [16,40]. ADA is an enzyme related to the immune system whose salivary concentrations could increase in different inflammatory stimuli, and in a proteomic study, this enzyme was one of the most upregulated proteins in pigs with diarrhea [40]. Aldolase is considered a marker of sepsis in pigs, increasing in animals infected by *S. suis* [15]. Also, Calp has been recognized as a marker of immune activation and also sepsis both in humans [41,42] and pigs [43]. Hp is a moderate acute-phase protein in pigs that increases due to inflammation [16]. sAA has been described to be increased to protect the organism against gastrointestinal pathogens [44]. The increases in LDH could reflect tissue damage [45].

The results of this report indicated differences in the selected analytes among the diseases studied. For example, pigs with ETEC had higher values of Hp and aldolase than pigs with *S. suis.* Also, AOPPs were significantly increased only in the ETEC group, which is in line with the increases observed in pigs after the experimental induction of a septic status by *E. coli* lipopolysaccharide administration [45]. AOPPs are a marker of protein oxidation due to an elevated production of reactive oxygen species [30], and the higher levels observed in ETEC could be due to metabolic acidosis as a consequence of bicarbonate loss due to diarrhea.

In our report, pigs with PRRS did not show high ADA values, probably due to the immune depression induced by PRRSV [14]. Also, the absence of high aldolase values could be related to the absence of sepsis, which could correspond with the lack of fever in the pigs with PRSS in our report. Hp, Calp, sAA, and LDH were unique biomarkers that showed higher values in pigs with PRRS than in healthy animals, probably due to the inflammation, stress, and tissue damage that these pigs might have suffered. Increases in salivary Hp have been described in pigs after experimentally induced PRRS [46]. In spite of the higher values of Calp found in the pigs affected by PRRS, they were lower than those observed in the two bacterial processes included in this study. Since lower increases in salivary Calp have been described in pigs with non-septic inflammation than in pigs with septic inflammation [43], this result could be in line with the absence of septic complications.

Once the different behaviors of analytes are verified according to the disease, the use of machine learning techniques can be useful for finding predictive algorithms that could be used as an aid to suspect or predict the probability of the presence of a specific disease. Thus, predictive models were constructed by BLR and DT analyses. The BLR analyses revealed that increased Calp and TP were associated with *S. suis* infection, but the accuracy obtained in the validation set was low and non-significant. The DT model, in contrast, considered ADA, TP, and sAA as significant variables. But similarly, as with BLR, the model did not effectively predict *S. suis* infection when it was applied to the validation set. Therefore, none of the models were able to effectively discriminate *S. suis* infection from other conditions (healthy, ETEC, and PRRS). Therefore, it could be postulated that the profile of saliva analytes can differentiate *S. suis*-infected pigs from healthy pigs, but not from pigs with other diseases.

In contrast to *S. suis*, the results of the predictive models were suitable for predicting pigs affected by ETEC infection and PRRS. High ADA and Hp values, together with low Calp, were related to ETEC, with similar accuracies in the validation set for the BLR and DT models (81.8% and 78.6%, respectively). On the other hand, increased LDH activity without an increase in ADA was related to PRRS, with the overall validation accuracies being very similar between the models (89.6% and 87.3% for BLR and DT, respectively). In practice, elevated values of Hp and ADA in the saliva could be indicative of ETEC in the animals from our study, whereas increased LDH activity and low ADA activity could be indicative of PRRS.

This study has some limitations that should be taken into account. Firstly, it is important to consider that these analyses in saliva cannot replace, in any case, either the clinical judgment made by the practitioner based on clinical signs or the use of molecular diagnoses of the specific pathogen. In addition, saliva can be contaminated by ambient debris, feed particles, or feces that can interfere with the measurements of analytes, and therefore non-contaminated samples should be used. However, the use of salivary analytes can be considered an easy and non-expensive aid in the context of a farm that is being monitored and in which a systematic and protocolized use of salivary biomarkers could be useful, both to detect an outbreak and to suspect the possible agent involved. In this line, with the exception of haptoglobin, the rest of the analytes in this report was measured by spectrophotometric assays and in an automated analyzer, and therefore they could be set up and used as part of the routine in clinical pathology laboratories. Secondly, the number of animals included in this study was low, so the study should be considered a pilot report to be replicated and corroborated with a wider population. Thirdly, the groups that were compared in this study did not share the same origin and age. This is important since PRRS commonly appears in older animals than *S. suis* or ETEC. In order to minimize the possible confounding factor of this variable, the age of the animals was included as a covariate in the BLR and ROC analyses. Regarding the interpretation of the results, some overlapping was observed between the groups of animals. Therefore, these models, especially in cases of bacterial diseases, had trouble differentiating between *S. suis* and ETEC. Also, other processes can affect some of the analytes, including stress or other non-infectious health issues. For instance, this is the case for Hp, which, like other markers of immunity and inflammation, can increase in stressful situations in this species [47]. Thus, the absence of confounding factors should be discarded for a proper interpretation of the biomarker results. Anyway, the absence of an increased level of one specific analyte could potentially be useful for discarding a specific process. In addition to these limitations, the panel of analytes considered in this study was limited, so it would be of great benefit to explore a wider number of analytes that could be more specific to a disease and potentially include them in the models, for example, creatine kinase and troponins, since they have shown some value in indicating the presence of *S. suis* infection [16].

Even though the presence of concomitant diseases was not discarded in the animals included in this study, none of the animals from the PRRS group showed fever that, together with the lack of high ADA and aldolase values, could indicate that a concomitant disease was likely not present. However, another possible limitation for the application of these findings is the presence of concomitant diseases. PRRSV has tropism by cells of monocytic [48,49] and lymphoid origins [50,51,52], subsequently affecting the host immunity response. As a consequence, coinfection with secondary pathogens is a common finding. Coinfection with *Mycoplasma hyopneumoniae* [53], *Bordetella bronchiseptica* [54], *Glaesserella parasuis* [55], or even with other viruses such as porcine circovirus type 2 [56], coronavirus, or swine influenza virus [57] results in more severe disease and respiratory symptoms. Also, PRRSV infection at weaning is considered a risk factor for concomitant *S. suis* infection [58]. The presence of some of these concomitant diseases would potentially affect the values of the salivary biomarkers, which would make their interpretation more complicated, and it should be further studied. Also, different lineages of PRRSV have been detected in Europe, with different geographic distributions, inducing clinical signs of different severities [59]. Therefore, the results obtained in this study should not be strictly extrapolated to other European regions or PRRSV strains.

## 5. Conclusions

In conclusion, salivary analytes showed different changes in pigs depending on the disease, with the highest ADA and Hp values in pigs with ETEC, whereas animals with PRRS showed lower ADA and higher LDH activities. The constructed predictive models using salivary analytes showed overall accuracies of over 78% and 87% for detecting ETEC and PRRS, respectively, in the conditions of this study. Since saliva analyses are an easy way to monitor animals, the implementation of these analyses in pig farms could be useful for detecting and predicting the presence of specific diseases. Further studies should be conducted in larger populations to confirm and refine these findings and evaluate their possible applications, and to explore additional analytes not studied in this report. These analytes can be measured by commercial laboratories, which would support the possible commercial use of this methodology if the results of this report are corroborated by additional large-scale studies.

## Figures and Tables

**Table 1:** Binary logistic regression (BLR) and decision tree (DT) analyses performed to predict one diseased group from the rest of the groups. For BLR, only those analytes with a significant area under the curve (AUC) and included in the equation are shown.

			AUC (ER)	95% CI	*p*-Value	Cut-Off	Sens%	Spec%	Acc%
*S. suis*	BLR	Calp	0.664 (0.050)	0.566–0.762	0.001	>0.40 mg/L	54.8	71.5	48.9
		TP	0.664 (0.047)	0.571–0.757	0.001	>180.83 mg/dL	70.2	60.3	65.3
		Training model	0.787 (0.048)	0.692–0.882	<0.001		67.9	81.9	74.9
		Validation model	0.676 (0.112)	0.456–0.825	0.135		71.4	58.0	64.7
	DT	Training model	0.668 (0.033)	0.603–0.734	<0.001		45.8	85.5	71.9
		Validation model	0.603 (0.057)	0.492–0.715	0.068		40.0	83.6	68.6
ETEC	BLR	ADA	0.722 (0.037)	0.649–0.796	<0.001	>4203.20 IU/L	79.6	63.9	71.8
		Hp	0.780 (0.040)	0.701–0.859	<0.001	>3426.69 ng/mL	87.2	60.7	74.0
		Training model	0.852 (0.034)	0.787–0.918	<0.001		76.9	84.0	80.4
		Validation model	0.884 (0.045)	0.795–0.972	<0.001		75.0	88.6	81.8
	DT	Training model	0.788 (0.035)	0.719–0.857	<0.001		53.7	95.7	86.3
		Validation model	0.682 (0.056)	0.571–0.792	0.001		39.1	90.0	78.6
PRRS	BLR	ADA	0.689 (0.036)	0.618–0.760	0.001	<6332.13 IU/L	100.0	48.0	60.5
		LDH	0.659 (0.049)	0.564–0.754	0.005	>273.60 IU/L	61.8	63.5	62.6
		Training model	0.838 (0.036)	0.767–0.908	<0.001		60.6	90.4	75.5
		Validation model	0.910 (0.036)	0.838–0.981	<0.001		94.1	85.1	89.6
	DT	Training model	0.838 (0.032)	0.776–0.900	<0.001		43.2	98.1	89.7
		Validation model	0.803 (0.048)	0.708–0.899	<0.001		26.7	97.7	87.3

ER, standard error; CI: confident interval; Sens: sensitivity; Spec: specificity; Acc: overall accuracy; ETEC: enterotoxigenic *Escherichia coli*; PRRS: porcine reproductive and respiratory syndrome; Calp: calprotectin; TP: total protein; ADA: adenosine deaminase; Hp: haptoglobin; LDH: lactate dehydrogenase.

## Data Availability

Dataset available on request from the authors due to privacy.
